# Levosimendan in patients undergoing extracorporeal membrane oxygenation after cardiac surgery: an emulated target trial using observational data

**DOI:** 10.1186/s13054-023-04328-6

**Published:** 2023-02-07

**Authors:** Julien Massol, Noémie Simon-Tillaux, Joanna Tohme, Geoffroy Hariri, Pauline Dureau, Baptiste Duceau, Lisa Belin, David Hajage, Yann De Rycke, Ahmed Charfeddine, Guillaume Lebreton, Alain Combes, Adrien Bouglé

**Affiliations:** Department of Anesthesiology and Critical Care Medicine, La Pitié-Salpêtrière University Hospital, 47-83 Boulevard de L’Hôpital, 75013 Paris, France

## Abstract

**Background:**

Retrospective cohorts have suggested that levosimendan may facilitate the weaning of veno-arterial extracorporeal membrane oxygenation (VA-ECMO). We therefore studied this clinical question by emulating a randomized trial with observational data.

**Methods:**

All patients with refractory postcardiotomy cardiogenic shock and assisted with VA-ECMO, admitted to a surgical intensive care unit at La Pitié-Salpêtrière Hospital between 2016 and 2019, were eligible. To avoid immortal-time bias, we emulated a target trial sequentially comparing levosimendan administration versus no levosimendan administration in patients treated with VA-ECMO. The primary outcome was time to successful ECMO weaning. The secondary outcomes were 30-day and 1-year mortality. We performed a multivariable analysis to adjust for confounding at baseline.

**Results:**

Two hundred and thirty-nine patients were included in the study allowing building a nested trials cohort of 1434 copies of patients. No association of levosimendan treatment and VA-ECMO weaning was found (HR = 0.91, [0.57; 1.45], *p* = 0.659 in multivariable analysis), or 30-day mortality (OR = 1.03, [0.52; 2.03], *p* = 0.940) and 1-year mortality (OR = 1.00, [0.53; 1.89], *p* = 0.999).

**Conclusions:**

Using the emulated target trial framework, this study did not find any association of levosimendan treatment and ECMO weaning success after postcardiotomy cardiogenic shock. However, the population of interest remains heterogeneous and subgroups might benefit from levosimendan.

**Supplementary Information:**

The online version contains supplementary material available at 10.1186/s13054-023-04328-6.

## Introduction

Veno-arterial extracorporeal membrane oxygenation (VA-ECMO) is the most widely used temporary mechanical circulatory support to restore adequate blood perfusion in patients with refractory cardiogenic shock [1–3]. Although VA-ECMO may improve the prognosis of patients with refractory cardiogenic shock, it remains a bridge therapy [2, 4]. Furthermore, the use of VA-ECMO is associated with specific serious complications and mortality increases with the duration of support [2, 5–10]. A critical aspect of the clinical management of patients on VA-ECMO after cardiac surgery is optimizing cardiac function to facilitate weaning from VA-ECMO [11]. Levosimendan (SIMDAX, Orion) is a calcium-sensitizing inotrope with systemic, coronary, and pulmonary vasodilator effects as well as specific cardioprotective effects [12, 13]. The use of levosimendan in patients under VA-ECMO appears interesting from a mechanistic point of view, particularly to reduce the duration of mechanical assistance and thus minimizing serious complications and maybe reducing mortality. Several clinical studies and meta-analyses have shown a benefit when it comes to weaning from VA-ECMO, and short and long-term survival, in various medical and surgical populations [14–16]. However, all of these studies are observational, may have suffered from immortal-time bias, and fail to take into account the competing risks for ECMO weaning success. We therefore sought to evaluate whether levosimendan administration may improve VA-ECMO weaning or may impact mortality in a large population of patients with postcardiotomy cardiogenic shock by using the emulated target trial framework in order to better control the aforementioned biases.

## Materials and methods

### Regulations and ethics

This is a single-center cohort study with retrospective data collection. Patients included in this study received standard of care without any change related to the research. Patient data were handled in accordance with the French regulations (MR004 reference methodology). The study protocol was approved by the Research Ethics Committee of the French Society of Anesthesiology and Critical Care Medicine (CERAR), registered as IRB number 00010254-2021-124. The reporting of this study complies with the STROBE statement [17]. The STROBE checklist is provided in Additional file 1: Table S1.

### Patient population

We enrolled all patients admitted to the surgical intensive care unit (ICU) at the Cardiology Institute of La Pitié-Salpêtrière University Hospital (Paris, France) between January 2016 and December 2019. The main inclusion criterion was the presence of mechanical circulatory support with VA-ECMO in the setting of cardiovascular surgery. We excluded patients who were less than 18 years old, those who had other types of mechanical circulatory support, those who had ECMO in a non-surgical context, and those who started ECMO support before surgery.

### ECMO implantation

ECMO was implanted either in the operating room, as a relay of cardiopulmonary bypass when patients presented low cardiac output refractory to adrenergic agonists, or postoperatively in ICU when patients presented a refractory cardiogenic shock. Implantation was always performed by cardiac surgeons. The initial ECMO flow rate was set between 80 and 100% of patient’s theoretical cardiac output to ensure perfusion while maintaining cardiac ejection.

### ECMO weaning

Patients were weaned from VA-ECMO according to a local protocol previously reported [18]. Briefly, ECMO weaning was considered when patients presented with an ECMO flow rate less than or equal to 1.5L/min a mean arterial pressure > 65 mmHg in the absence or at low doses of vasopressors (< 0.1 µg/kg/min of norepinephrine), a pulsatile arterial waveform arterial, left ventricular ejection fraction (LVEF) > 25- 30%, subaortic time–velocity integral (TVI) > 12 cm, arterial partial pressure of oxygen (PaO2)/fraction of inspired oxygen (FiO2) > 200, with FiO2 delivered by the extracorporeal circuit < 40% and that delivered by the ventilator circuit < 60%.

### Levosimendan administration

All patients in the levosimendan group received a continuous infusion of 0.2 µg/kg/min over 24 h (i.e., 20 mg for a patient of 70 kg) administered as a single dose without an initial bolus of levosimendan according to local standard protocol. Administrated levosimendan was diluted in 0.9% NaCl at a concentration of 0.25 mg/mL, according to the manufacturer recommendations. In this study, none of the patients who received levosimendan exhibited adverse effects requiring cessation of the drug administration before completion of the standard dose.

### Outcomes definition

The primary outcome was time to successful weaning from VA-ECMO within 30 days. Successful ECMO weaning was defined as follows: ECMO removal within 30 days in a patient being alive and without one of the following events occurring in the 30 days after ECMO removal: death, or need for repeated ECMO support or another mechanical circulatory device or heart transplantation (defining the ECMO weaning failure after ECMO removal). The secondary outcomes were 30-day mortality and 1-year mortality.

### Data collection

Baseline covariates and follow-up of patients were collected retrospectively by medical record consultation and computer extraction of biological data. The following covariates were collected at baseline: age, height, weight (with calculation of body surface area according to Boyd's formula), sex, Simplified Acute Physiology Score (SAPS) II at ICU admission, history of chronic left heart failure, hypertension, previous sternotomy, chronic kidney disease, diabetes mellitus, indication for surgery, and type of acute heart failure at beginning of ECMO support. The following covariates were collected daily during the ICU stay: creatinine, total bilirubin, platelet count, ECMO output, ongoing renal replacement therapy (RRT), lactate, total epinephrine dose, total norepinephrine dose, and total dobutamine dose. If multiple values were available for a covariate, the maximum value for the day was retained, except for platelet count, for which the minimum value was retained.

The daily biological SOFA score was calculated from the daily values of creatinine, platelet count, and total bilirubin, as described elsewhere [19]. Similarly, daily vasoactive inotropic score (VIS) was calculated from the total daily dose of epinephrine, norepinephrine, and dobutamine [20].

### Target trial emulation framework

In this study, we aimed to evaluate whether levosimendan administration in patients with VA-ECMO support shortened the time to successful ECMO weaning and reduced mortality at 30-day and at 1-year. We thus emulated a hypothetical target trial in which patients were randomly assigned to levosimendan administration or no levosimendan administration, and for which eligibility criteria, ECMO procedures (implantation and weaning), and outcomes would be the same for its emulation with observational data [21–23]. See Additional file 1: File, Table S2, and supplemental references for more information on the target trial emulation framework and its specification in this study.

**Fig. 1:**
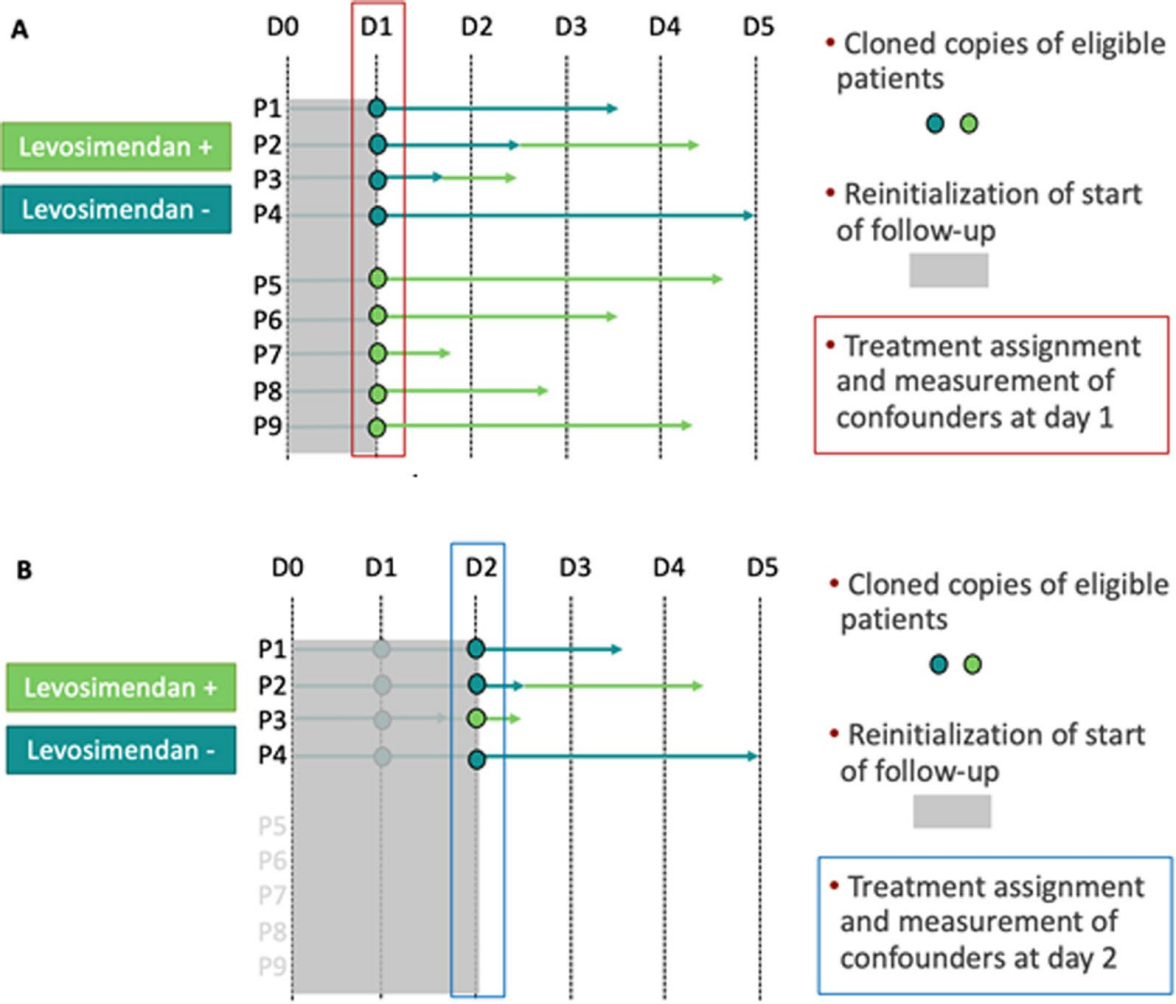
Sequential emulation of the target trial. The target trial (as defined in Additional file 1: Table S2) was emulated several times in a sequence of nested trials, to make coincide treatment assignment and start of follow-up, thus avoiding immortal-time bias. This figure describes the construction of the observations in each nested trial with their respective treatment assignment, measurement of confounders, and follow-up. A hypothetical cohort is presented for simplicity. Panel** A**: The figure depicts the construction of the first emulated trial. At day 0 (first day of ECMO support), no patient received levosimendan; thus, the target trial was not emulated as the comparison was not feasible between patient receiving and not receiving levosimendan. At day 1 (D1), five patients (P5 to P9) started levosimendan, and so could be compared to the four patients (P1 to P4) that did not receive levosimendan. Their treatment assignment is in line with the received strategy at the day of the target trial emulation (here day 1). In addition, baseline confounders for the first nested trial are measured on day 1 (red rectangle), that is, day 1 since beginning of ECMO. Follow-up is restarted at day 1 for the first emulated trial to make coincide eligibility, treatment assignment and time-zero. Panel** B**: The figure depicts the construction of the second emulated trial. At day 2 after beginning of ECMO support, three patients did not receive levosimendan (P1, P2, and P4) and one patient received levosimendan (P3). Patients that received levosimendan in the previous nested trial at day 1 are not eligible anymore, as defined in the target trial protocol (new-user design). As for day 1 nested trial, treatment assignment of cloned copies of eligible patients is based on the treatment received the day of the emulated trial (blue rectangle) and baseline confounders for the second nested trial are measured on day 2. Follow-up is again restarted on the day 2 of the nested trial. Each of these nested emulated trials data is then stacked in a unique dataset, built with cloned copies of participants with assigned treatment, reset follow-up and baseline confounders in each nested trial. A multivariable Cox model is then fitted on the stacked cohort of observations and adjusted for confounders measured at ICU admission and at baseline of each nested trial (lactatemia, ECMO output, VIS, and SOFA score)

The target trial was emulated from our cohort each time the comparison between the two arms of the target trial were possible during the duration of ECMO support, i.e., when there was at least one patient eligible to the study who received levosimendan. Patients were eligible multiple times during follow-up; thus, a copy of a patient was created each time a patient could participate to a nested emulated trial. Patients eligible at a given time point were alive, still receiving ECMO, and did not receive levosimendan at previous time points (new-users design). At baseline of each emulated trial, patients were classified as receiving levosimendan if they started levosimendan on the ECMO day or not receiving levosimendan otherwise, and potential confounding was measured. The day zero (first follow-up) was defined as the day of beginning of ECMO support. Patients (and their respective copies) were followed from baseline of each emulated trial until death or 1-year after ICU admission (end of data collection on December 31, 2020). This methodological strategy makes coincide the time of eligibility criteria assessment, treatment assignment and starting of follow-up, thus removing immortal-time bias related to the delay between ECMO initiation and levosimendan administration [24]. For more information on the construction of the nested trials cohort, see Fig. 1.

### Description of the study populations

For each analytical population, patient selection process was represented by a flow diagram, and characteristics at inclusion were described as percentage for categorical variables, median with interquartile range (IQR), or mean with standard deviation (SD) for continuous variables, as appropriate. Characteristics were stratified according to whether levosimendan was administered for weaning from ECMO. The two groups were compared using adequate tests according to the type of variables and their distribution (Chi2 tests, Fisher’s exact tests, Wilcoxon's rank sum tests, or two-sample t tests).

### Primary outcome analysis

The time to successful weaning from VA-ECMO was assessed for each copy of patient. Follow-up started at beginning of ECMO support and ended at first ECMO removal or death. The 30-day period after ECMO removal was only used to qualify ECMO weaning success but not included in the calculation of the time-to-event. Patients that were still on ECMO support at 30 days were censored at 30 days. As the primary objective of the study was to estimate the etiologic effect of levosimendan on ECMO weaning success, we first took into account competing risk of death and of ECMO weaning failure after ECMO removal by using a cause-specific hazard model, as proposed by Austin, Lee, and Fine [25]. In the setting of a pragmatic trial, we first estimated the observational analog of a per-protocol effect [26]. To mimic this per-protocol population, observations were additionally censored at the date of levosimendan administration for patients who received levosimendan during follow-up but whose cloned copy had not yet started levosimendan, or the date of the second levosimendan administration for patients who received levosimendan twice. Because of informative censoring introduced by design in the control group, a strategy of inverse probability of censoring weighting (IPCW) was implemented, see Additional file 1: supplemental file for more information [27, 28]. For the analog of the intention-to-treat effect, these same patients were not censored during follow-up if they were treated with levosimendan after baseline assessment in each nested trial.

The effect of levosimendan on the primary outcome was then estimated on pooled nested trials data using a univariable and a multivariable Cox model to adjust for confounders measured at baseline of each nested trial, as the decision to prescribe levosimendan was at the discretion of the medical team in this cohort, and some confounders may be imbalanced between the two treatment groups at baseline of nested trials. Covariates entered into the model were as follows: age, sex, SAPS II at ICU admission, history of chronic heart failure, previous sternotomy, hypertension, diabetes mellitus, type of acute heart failure, indication for surgery, chronic kidney disease, BMI, SOFA score, lactatemia, ECMO blood flow rate indexed to body surface area, and VIS. SOFA score, ECMO output, lactatemia, and VIS were measured at baseline of each nested trial, all other covariates were measured at ICU admission or at beginning of ECMO support. All confounders were entered into the model based on clinical expertise and prior knowledge [2, 15, 29–31]. Because of violation of the proportional hazards assumption, some covariates were transformed to get time-varying effects; see Additional file 1: supplemental file for more information.

Mortality at 30 days and at 1 year after assignment to the nested trials was estimated in a univariable analysis by calculating the odds ratio and its confidence interval. Multivariable analyses with logistic regression were then performed to adjust for confounders. See Additional file 1: supplemental file for more information*.*

### Sensitivity analyses

First, we performed a sensitivity analysis excluding patients from 2016, as only four patients received levosimendan this particular year (9% of total of patients eligible to our study in 2016), whereas the proportion of patients receiving levosimendan in 2017, 2018, and 2019 was quite stable (comprised between 26 and 38%), and to assess whether change in clinical practice may have affected the per-protocol estimate of effect of levosimendan on the primary outcome. Then, we used Fine and Gray’s competing risks model to estimate the subdistribution hazard ratio for ECMO weaning success (sHR) [32]. The multivariable competing risks model was adjusted on the same covariates than the multivariable Cox model previously described (see Additional file 1: supplemental file for more information).

### Subgroup analyses

Several subgroups were explored for the primary outcome, with tests for treatment-by-subgroup interaction. The interaction terms tested were: time to treatment assignment, or the effect of an early levosimendan administration versus a later one after beginning of VA-ECMO support (until day 2 or after day 2), time of ECMO implantation (in the operating room or in the ICU), type of acute heart failure (right or left ventricular heart failure or right and left ventricular heart failure), indication for surgery (heart transplantation versus others), and renal replacement therapy (RRT) at baseline (ongoing dialysis on the day of the nested trial). The interaction was tested with the Wald test.

### Missing data handling

Missing data for all covariates were handled with an approach that considers the time-dependence of the covariates in the imputation model [33, 34]; see Additional file 1: supplemental file for more information. No patient was lost to follow-up for the primary outcome (including the additional 30-day time for qualifying the event) and 30-day mortality. Due to a low proportion of missingness (less than 3%) and their exploratory nature, the analyses were performed on complete cases for 1-year mortality.

Robust variances were estimated by bootstrap (1000 iterations) to account for the fact that observations of patients have been duplicated in the analysis. Significance level was set at 0.05. Analyses were performed using R software, version 4.1.2 (R Core Team 2021, http://cran.rproject.org) using the following packages: survival (v3.2–13, [35]), mice (v3.14.0, [36]), and cmprsk (v2.2–10, [37]).

## Results

### Population included at the beginning of ECMO support

**Fig. 2:**
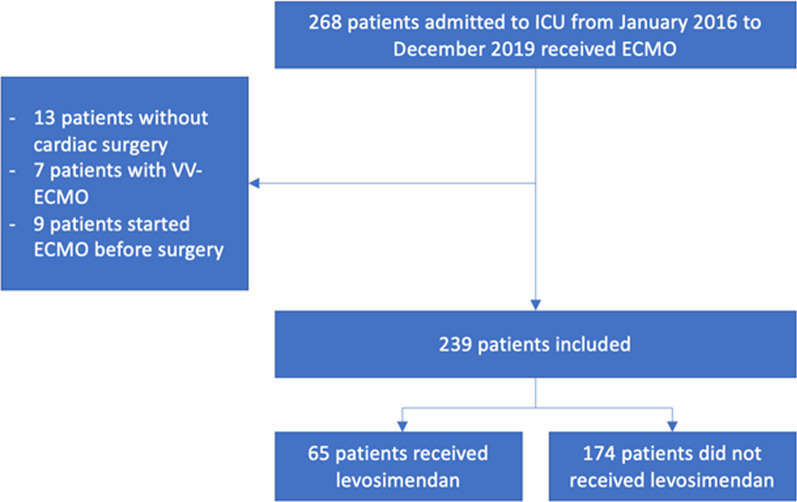
Flow diagram of the population included at the beginning of ECMO support

Between January 1, 2016, and December 31, 2019, 239 patients were included in the study, among whom 65 received levosimendan (flowchart, Fig. 2). Patients who received levosimendan had a lower BMI (median 25, first and third quartiles Q1–Q3 [22–28] versus 27, Q1–Q3 [24–31], *p* = 0.015), higher SAPS 2 score and Euroscore at admission (median 55.5, Q1–Q3 [12.65–64.5] versus 24, Q1–Q3 [5–51], *p* = 0.0004, and median 6.44, Q1–Q3 [2–25] versus 3.85, Q1–Q3 [2–10.3], *p* = 0.047, respectively), more often isolated left ventricular failure at ECMO implantation and less often biventricular failure (22/65 (33.8%) vs. 28/174 (16.1%) and 35/65 (53.8%) vs. 126/174 (72.4%), respectively, *p* = 0.008), and received higher doses of daily dose of dobutamine at beginning of ECMO support (median 365.5 mg, Q1–Q3 [28.1–615.6] versus 226.35 mg, Q1–Q3 [0–517.35], *p* = 0.041). During follow-up, patients who received levosimendan had more strokes (13/65 (20.0%) vs. 13/174 (7.5%), *p* = 0.006) and less acute mesenteric ischemia (5/65 (7.8%) vs. 37/174 (21.5%), *p* = 0.014). Population’s characteristics are summarized in Table 1.

**Table 1:** Description of the population included at the beginning of ECMO support

			Levosimendan		*p*-value
			No (*n* = 174)	Yes (*n* = 65)	
**On admission**					
Age (years)	Median [Q1–Q3]		62 [54–70]	62 [58–72]	0.725
Sex	Female		67 (39%)	22 (34%)	0.507
BMI (Kg/m^2^)	Median [Q1–Q3]		27 [24-31]	25 [22–28]	0.015
	N (NA)		173 (1)	65 (0)	
Medical conditions	Hypertension		99 (57%)	36 (55%)	0.834
	Diabetes		40 (23%)	20 (31%)	0.217
	Dyslipidemia		59 (34%)	26 (40%)	0.381
	COPD		18 (10%)	6 (9%)	0.799
	Peripheral vascular disease		16 (9%)	9 (14%)	0.278
		N (NA)	175 (0)	64 (1)	
	Chronic heart failure		131 (78%)	49 (77%)	0.758
		N (NA)	168 (7)	64 (1)	
	Previous sternotomy		67 (39%)	20 (31%)	0.269
	Chronic kidney disease		49 (28%)	18 (28%)	0.943
SAPS II	Median [Q1–Q3]		24 [5–51]	56 [13–64]	0.0004
	N (NA)		173 (1)	64 (1)	
Euroscore	Median [Q1–Q3]		3.9 [2–10.3]	6.4 [2-25]	0.047
	N (NA)		172 (8)	64 (4)	
Type of surgery	CABG		13 (7%)	10 (15%)	0.262
	Valvular surgery		55 (32%)	18 (28%)	
	Combined valvular and CABG		20 (11%)	4 (6%)	
	Heart transplant		65 (37%)	26 (40%)	
	LVAD		0 (0%)	1 (2%)	
	TAVI		1 (1%)	0 (0%)	
	Other		20 (11%)	6 (9″)	
**In the operating room**					
CPB duration (minutes)	Median [Q1–Q3]		142 [110–180]	136 [98–190]	0.463
	N (NA)		172 (2)	65 (0)	
Aortic cross-clamping (minutes)	Median [Q1–Q3]		85 [67–124]	77 [55–127]	0.292
	N (NA)		163 (11)	62 (3)	
RBC (units)	Median [Q1–Q3]		2 [0–6]	2 [0–5]	0.552
	N (NA)		169 (5)	64 (1)	
Frozen plasma (units)	Median [Q1–Q3]		3 [0–6]	2 [0–6]	0.224
	N (NA)		169 (5)	64 (1)	
Platelets (units)	Median [Q1–Q3]		1 [0–1]	1 [0–1]	0.660
	N (NA)		169 (5)	64 (1)	
Fibrinogen	Yes		57 (34%)	20 (31%)	0.741
	N (NA)		171 (4)	64 (1)	
**At ECMO implantation**					
Timing	Intraoperative		126 (72%)	51 (78%)	0.3425
	Postoperative		48 (28%)	14 (22%)	
Ventricular failure at implantation	Left		28 (16%)	22 (34%)	0.008
	Right		20 (11%)	8 (12%)	
	Right and left		126 (72%)	35 (54%)	
ECMO site	Central		13 (7%)	3 (5%)	0.568
SOFA	Median [Q1–Q3]		11 [10–13]	11 [10–12]	0.341
	N (NA)		136 (38)	57 (8)	
Daily total dose of epinephrine (mg)	Mean (SD)		13 (27.9)	6.8 (15.2)	0.070
	N (NA)		172 (2)	65 (0)	
Daily total dose of norepinephrine (mg)	Median [Q1–Q3]		17.6 [6.2–42.2]	15.4 [4.1–38.1]	0.312
	N (NA)		172 (2)	65 (0)	
Daily total dose of dobutamine (mg)	Median [Q1–Q3]		226.3 [0–517.3]	365.5 [28.1–615.6]	0.041
	N (NA)		172 (2)	65 (0)	
VIS	Median [Q1–Q3]		26.8 [13.3–52.9]	23.6 [13.8–40.4]	0.275
	N (NA)		172 (2)	65 (0)	
Lactate	Median [Q1–Q3]		5.6 [3.4–8.9]	5.3 [3–7.9]	0.160
	N (NA)		157 (17)	59 (6)	
ECMO output (L/min/m2)	Median [Q1–Q3]		1.8 [1.5–2.1]	1.9 [1.4–2.2]	0.686
	N (NA)		131 (43)	55 (10)	
Impella®	Yes		12 (7%)	4 (6%)	1.000
Delay between CPB cessation and ECMO implantation (hours)	Mean (SD)		8.4 (26.4)	7.4 (28.5)	0.295
	N (NA)		163 (11)	64 (1)	
Delay between ECMO implantation and levosimendan infusion (days)	Median [Q1–Q3]		-	5 [3–6]	
**Events under ECMO**	IABP		73 (42%)	27 (42%)	0.954
	Digestive bleeding		25 (14%)	13 (20%)	0.289
	Acute mesenteric ischemia		37 (22%)	5 (8%)	0.014
		N (NA)	173 (2)	64 (1)	
	Acute kidney injury		136 (79%)	44 (68%)	0.0804
		N (NA)	174 (1)	64 (1)	
	Extra-renal replacement therapy		96 (55%)	33 (51%)	0.543
	Stroke		13 (7%)	13 (20%)	0.006
	Mediastinitis		15 (9%)	10 (15%)	0.128
	Bacteremia		52 (30%)	27 (42%)	0.088
	Septic shock		85 (49%)	36 (55%)	0.369
	Hemorrhagic shock		83 (48%)	30 (46%)	0.831
	Cardiac arrest		29 (17%)	8 (12%)	0.407
	Acute coronary syndrome		14 (8%)	1 (2%)	0.076
	VAP		110 (63%)	47 (72%)	0.188
	Limb ischemia		25 (15%)	8 (13%)	0.708
		N (NA)	172 (3)	63 (2)	
	Scarpa infection		18 (11%)	8 (13%)	0.650
		N (NA)	161 (4)	63 (2)	
	Surgical revision of the ECMO implantation site		26 (15%)	10 (16%)	0.937
		N (NA)	172 (3)	64 (1)	

BMI, body mass index; CABG, coronary artery bypass graft; CPB, cardiopulmonary bypass; COPD, chronic obstructive pulmonary disease; ECMO, extracorporeal membrane oxygenation; IABP, intra-aortic balloon pump; LVAD, left ventricular assist device; NA, not available (missing data); Q1, first quartile; Q3, third quartile; RBC, red blood cells; SAPS II, Simplified Acute Physiology Score II; SOFA, Sequential Organ Failure Assessment; TAVI, transcatheter aortic valve implantation; VAP, ventilator-associated pneumonia; VIS, vasoactive inotropic score. For covariates which the median was null in both groups, they were described with their mean and standard deviation

### Outcomes of the population included at the beginning of ECMO support

Levosimendan was administered after a median of 5 days after starting ECMO, Q1–Q3 [3–6]. The duration of ECMO was longer in the levosimendan group (median duration of 11 days, Q1–Q3 [7–16] versus 6 days, [3.25–9]), as well as the length of the ICU stay (median duration of 18 days, Q1–Q3 [14–33] versus 13 days, Q1–Q3 [6–23.5]). Patients were more often successfully weaned from ECMO in the levosimendan group (39/65 (60.0%) versus 81/174 (46.6%)) at the end of follow-up. Death within 30 days following ICU admission occurred in 19/65 patients (29.3%) in the levosimendan group and 79/174 (45.4%) in the control group. Death within one year following ICU admission occurred in 27/60 patients (45.0%) in the levosimendan group and 94/172 (54.7%) in the control group. After one year, seven patients were lost to follow-up.

### Target trial emulation

**Fig. 3:**
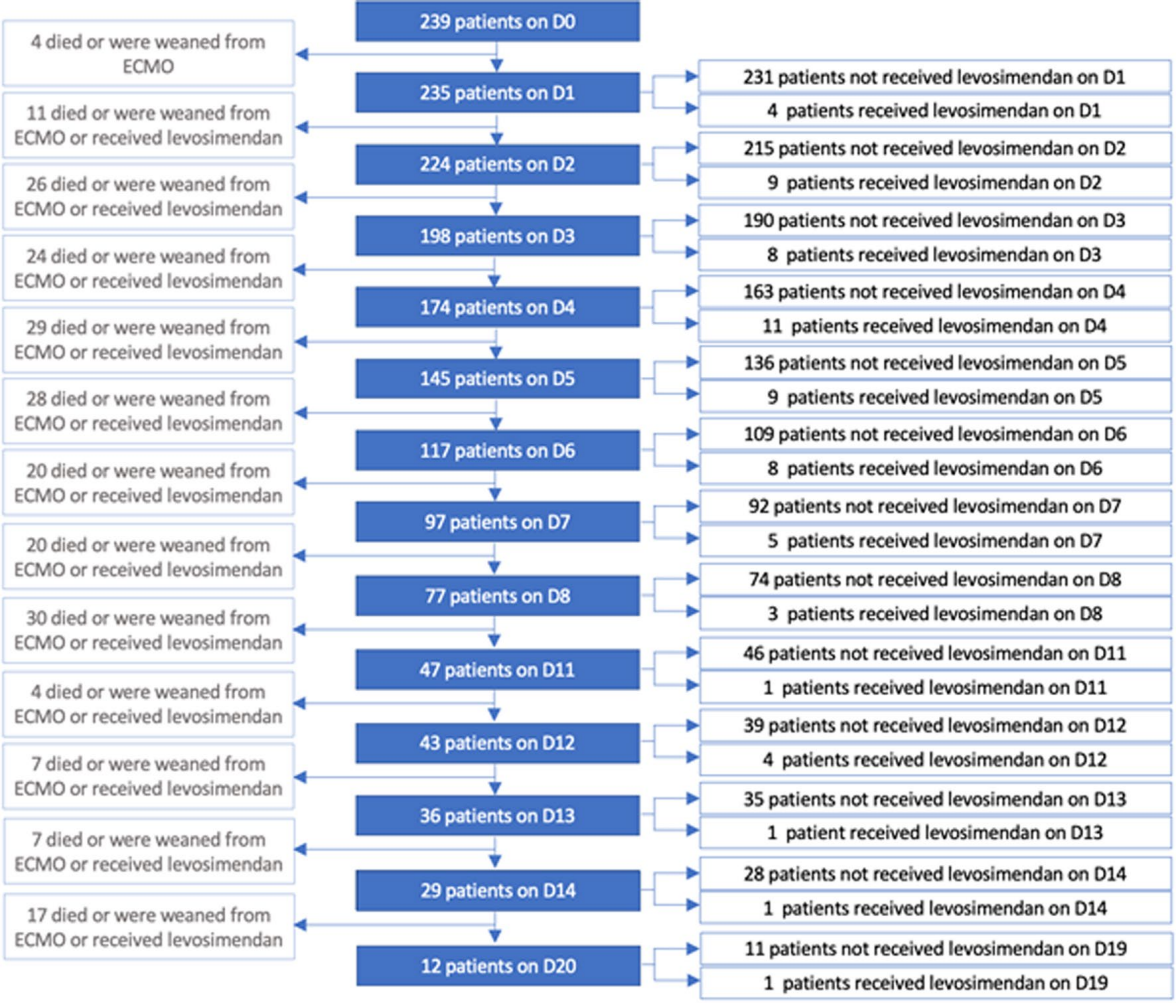
Flow diagram of the nested trials cohort. A nested trial was emulated each day (D) a patient received levosimendan since beginning of ECMO support

A total of 65 patients were included in the levosimendan group and 1369 copies of patients in the control group. The description of the target trial emulation population construction is shown in the flowchart in Fig. 3. The cumulative incidence of levosimendan prescription as a function of ECMO duration is shown in Additional file 1: Fig. S1. Target trials were emulated each day after ECMO implantation from day 1 to day 8, from day 11 to day 14, and at day 20.

In the nested trials population, patients receiving levosimendan had lower BMI (median 25, Q1–Q3 [22–28] versus 27, Q1–Q3 [23–31], *p* = 0.011), more often isolated left ventricular failure and less frequently biventricular failure (22/65 (33.9%) versus 237/1369 (17.3%) and 35/65 (53.9%) versus 984/1369 (71.9%), respectively, *p* = 0.002), and higher SAPS 2 on admission (median 55.5, Q1–Q3 [12.65–64.5] versus 34.1, Q1–Q3 [4.88–57], *p* = 0.001). They had a lower daily biological SOFA (median 11, Q1–Q3 [10–12] versus 12, Q1–Q3 [11–13], *p* = 0.005), a lower lactate (median 1.4, Q1–Q3 [1.1–2] versus 1.7, Q1–Q3 [1.1–2.7], *p* = 0.013), lower doses of epinephrine (mean total daily dose 4.7 mg, standard deviation (SD) 15.09 versus 1.35 mg, SD 6.19, *p* = 0.02) and dobutamine (median 0, Q1–Q3 [0–360.1] versus 186.6, Q1–Q3 [0–593.35], *p* = 0.002), and lower VIS score (median 5.85, Q1–Q3 [0.77–12.18] versus 8.84, Q1–Q3 [4.02–25.87], *p* = 0.002). During follow-up, the population of the levosimendan group had less acute mesenteric ischemia (5/64 (7.8%) versus 258/1350 (19.1%), *p* = 0.023), acute kidney injury (44/65 (67.7%) versus 1117/1361 (82.1%), *p* = 0.004), see Additional file 1: Table S3.

### Outcomes

**Fig. 4:**
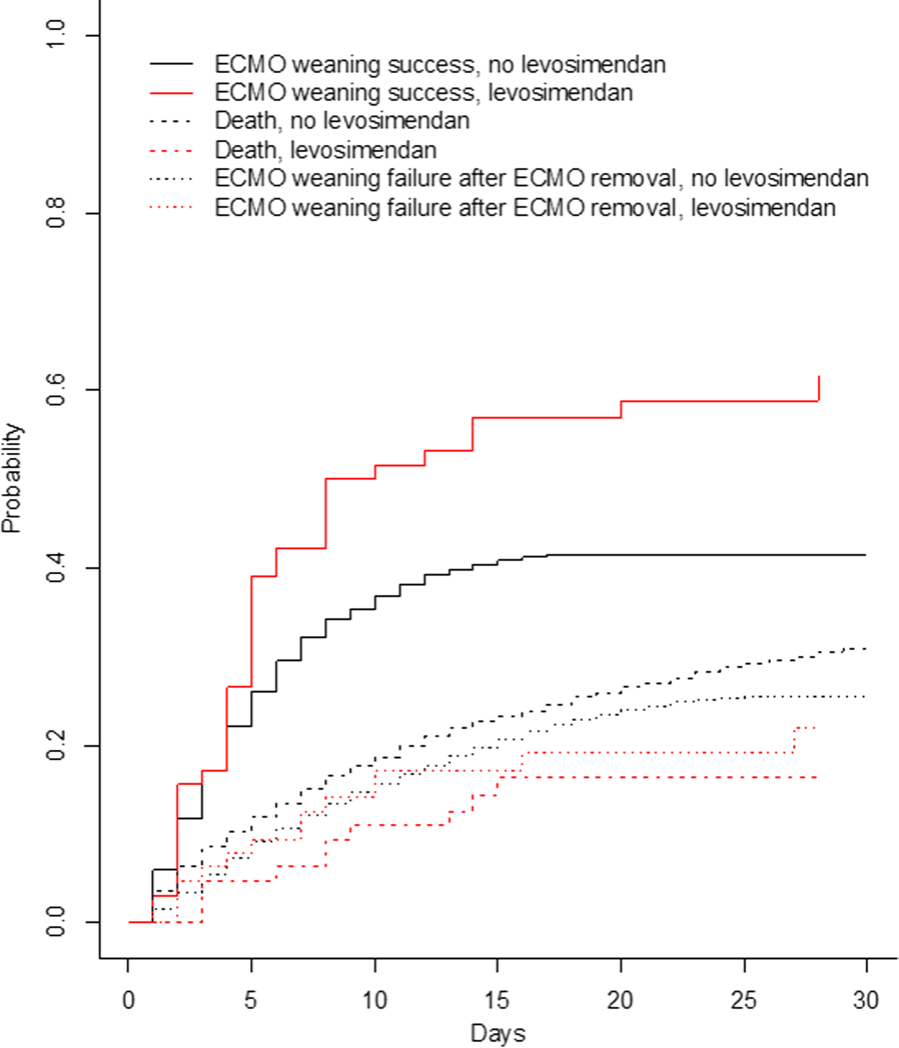
Cumulative incidences of ECMO weaning success in the presence of competing risks of death and ECMO weaning failure after ECMO removal. The cumulative incidence curves of ECMO weaning success were plotted with the Gray’s estimator that accounts for the competing risk of death and the competing risk of ECMO failure after ECMO removal [32]. The x-axis represents the days since the beginning of ECMO support

Regarding the time to successful ECMO weaning, no statistically significant association with levosimendan treatment was found in univariable analysis (cause-specific hazard ratio HR = 1.34, 95% confidence interval CI95 [0.92; 1.96], *p* = 0.122, in the analog of the per-protocol dataset) in the cause-specific hazard model for competing risks. Likewise, in multivariable analysis, levosimendan was not associated with successful ECMO weaning (HR = 0.91, CI95 [0.57; 1.45], *p* = 0.659), see Additional file 1: Table 4. The effect of levosimendan on ECMO weaning success was also non-significant after exclusion of patients from 2016, (adjusted HR of 0.73, CI95 [0.44; 1.21], per-protocol estimate). Sensitivity analysis with the subdistribution hazard competing risk model found in univariable analysis a statistically significant association between levosimendan treatment and successful ECMO weaning (subdistribution hazard ratio sHR = 1.60 CI95 [1.1; 2.32], *p* = 0.014) that was not significant anymore after adjustment on confounding factors (sHR = 0.99, CI95 [0.64,1.54], *p* = 0.973). Cumulative incidences curves for ECMO weaning success and competing risks of death and ECMO weaning failure are shown in Fig. 4. Analog of the intention-to-treat effect for the primary outcome was very similar compared with the previous analog of per-protocol estimate (HR = 0.98, CI95 [0.71; 1.35], *p* = 0.889).

Subgroup analyses according to the timing of ECMO implantation, timing of treatment assignment (levosimendan received in the first three days of ECMO or after), single or biventricular failure, ECMO implantation timing (in the operating room or in the ICU), indication for surgery, and RRT showed no significant difference between patients receiving levosimendan or not (Table 2).

**Table 2:** Subgroups analyses for the effect of levosimendan on ECMO weaning success: multivariable Cox model

	HR [CI95]	*p* for interaction
ECMO insertion in the OR	0.82 [0.49; 1.38]	0.579
ECMO insertion in ICU	1.29 [0.28; 5.91]	
Treatment assignment days 0–2	1.29 [0.42; 3.92]	0.474
Treatment assignment after day 2	0.85 [0.51; 1.40]	
Monoventricular heart failure	1.13 [0.50; 2.57]	0.593
Biventricular heart failure	0.86 [0.48; 1.53]	
Heart transplantation	0.73 [0.36; 1.49]	0.342
Others indications for surgery	1.12 [0.63; 2.00]	
No RRT	0.85 [0.53; 1.36]	0.932
RRT	1.07 [0.25; 4.54]	

CI95, 95% confidence interval; ECMO, extracorporeal membrane oxygenation; HR, hazard ratio; ICU; intensive care unit; OR; operating room; RRT: renal replacement therapy at baseline of the nested trial. Robust variances were estimated by bootstrap (1000 iterations)

For the secondary outcomes, there was no significant association of levosimendan and 30-day mortality in univariable (OR = 0.60, CI95 [0.35; 1.03], *p* = 0.064) nor multivariable analysis (OR = 0.99, CI95[0.51,1.94], *p* = 0.987). Likewise, there was no significant association of levosimendan and 1-year mortality in univariable (OR = 0.65, CI95 [0.39; 1.08], *p* = 0.098) nor multivariable analysis (OR = 1.00, CI95 [0.53; 1.91], *p* = 0.995).

## Discussion

Our study did not find any association of levosimendan administration and ECMO weaning success or mortality in patients with refractory postcardiotomy cardiogenic shock. To our knowledge, this is the first emulated target trial evaluating the potential effect of levosimendan on ECMO weaning and mortality in this population.

These results are consistent with the latest observational studies [15, 16] that did not found any impact of levosimendan on withdrawal or mortality. However, they are at odds with the trend in cohort studies on this topic, synthesized in the meta-analyses by Kaddoura et al. [14] and Burgos et al. [38] which concluded that there is a potential benefit of levosimendan on ECMO weaning and short- and long-term mortality in patients undergoing VA-ECMO for refractory cardiogenic shock.

Our study shows a number of strengths. The methodological framework known as "emulated target trial" [24] has advantages over the methods used in the studies published to date. First, if applied properly, it allows overcoming biases and particularly immortal-time bias, which is often present in pharmaco-epidemiological studies. In our study, the treatment assignment coincided with the assessment of eligibility criteria, and the beginning of follow-up. In previous studies, patients in the levosimendan group had at least survived until the administration of levosimendan, introducing an immortal-time bias [30]. Second, all studies published to date regarding the effect of levosimendan on VA-ECMO weaning did not estimate the effect of levosimendan for ECMO weaning success in presence of the concurrent risk of death and failure of ECMO weaning due to events that occurs after ECMO removal. Our analyses for the primary outcome accounting for competing risks were consistent in the cause-specific and subdistribution hazard models, thus strengthening the confidence in the results [32, 39]. Previous studies restricted the definition of ECMO weaning success to a very short timeframe after ECMO removal and may have overestimate the occurrence of this event. In this study, we use a more adequate definition of successful ECMO weaning, in line with the very long-lasting effects of levosimendan [40] and the poor prognosis of patients who received ECMO support during hospitalization, even after ECMO removal [41].

To date, all studies regarding the effect of levosimendan on ECMO weaning have been retrospective cohort studies and therefore all treatment effect estimates are at risk of confounding. Few studies [15, 30] assessed the effect of levosimendan after group matching on a propensity score; Distelmaier et al. [29] and Haffner et al. [42] estimated the potential effect of levosimendan in a multivariable analysis; all others studies did not adjust for measurable confounders. Our large cohort allowed us to introduce more explanatory covariates based on most recent literature [2] compared with previous studies.

Our study also has some limitations. First, even if we used the target trial framework, the study gets some limitations due to its observational nature: data were collected retrospectively, available information restrain the possibilities to define eligibility criteria, and blinding could not be emulated (even if in the setting of this particular study, we would have expected a minimal risk of performance and information biases). Second, missingness was present. Nonetheless, the proportion of missing data was low, and we imputed these data with multiple imputation, in a way that takes advantage of longitudinal collection of the data [33]. Third, although we estimated the association of levosimendan and ECMO weaning success adjusting on a large number of explanatory covariates, unmeasured confounders can still explain the observed estimates that would have been absent in a randomized trial. Most notably, we did not have heart transplant data for transplanted patients, who represent almost 40% of the study population. Fourth, despite the relatively large size of our cohort, and even if the nested trials design artificially increased the sample size of the control group, the power of analyses was still restricted by the limited number of observations that received levosimendan. Additionally, we did not find any subgroup with significant positive or negative possible effect of levosimendan on ECMO weaning.

Fifth, the dosage of levosimendan administered in our study (0.2 µg/kg/min) is in accordance with the label dosage (maximum dosage). To our knowledge, the only pharmacological study of levosimendan under ECMO by Sangalli et al. [43] found a benefit of levosimendan on endothelial function and cardiac output for doses twice as low as in our study. In our study, the maximum dosage was associated with hypotension requiring vasopressor support. Thus, the ideal dose may be lower. We look forward to the results of the “ECMO PK project” by Shekar et al. [44] regarding the pharmacokinetic changes under ECMO.

Sixth, the main cause of refractory postcardiotomy cardiogenic shock in our study was acute primary graft dysfunction. Therefore, the results of our study should be cautiously applied to other tertiary centers with more prevalent subpopulations at higher risk of failure of ECMO weaning (valvular, aortic and combined surgery) than patients after heart transplantation [2].

Finally, we could not control timing of administration of levosimendan as in a randomized trial, and the time between ECMO implantation and levosimendan administration was a median of 5 days [3–6]. This delay could influence weaning. Immohr et al. [45] found that, for heart transplant patients, early administration (< 48 h) was associated with faster weaning and a lower mortality in univariable analysis, with a trend toward reduced mortality in multivariable analysis. In our study, we did not find any effect modification related to the time between ECMO initiation and levosimendan administration in subgroup analysis. However, the delay between ECMO implantation and levosimendan infusion in the two studies finding a benefit of levosimendan on ECMO weaning was 1 day (Distelmaier et al. [29]) and 3.2 days (Vally et al. [30]) compared to 6.6 days in the study conducted by Guilherme et al. [15], thus very similar to our study, and in which no significant association of levosimendan and ECMO weaning was found.

The positive inotropic effect of levosimendan is prolonged because of 80-h half-life of the active metabolite OR-1896 [40]. Patients receiving levosimendan early may benefit from the pleiotropic effects of levosimendan in the early phase of ECMO, especially for protection against ischemia–reperfusion injury [13], while keeping benefit from this duration of effect to manage withdrawal.

## Conclusion

Our study did not find an association of levosimendan administration and VA-ECMO-weaning or mortality in patients with refractory postcardiotomy cardiogenic shock. These results issued from one of the largest cohorts studied to date and the only one using the emulated target trial approach might cast some doubt on the efficacy of levosimendan. The comparison with the results of an ongoing interventional study (LEVOECMO trial, NCT04728932) for the same clinical question may help to resolve the current discrepancies in observational data.

## Supplementary Information

**Additional file 1.** Supplemental tables and figure. Additional information for statistical methods.

## Data Availability

The datasets used and analyzed during the current study are available from the corresponding author on reasonable request.
