# Supplementary material for: Levosimendan in patients undergoing extracorporeal membrane oxygenation after cardiac surgery: an emulated target trial using observational data
Source: Crit Care. 2023 Feb 7;27:51. doi: 10.1186/s13054-023-04328-6 (PMC9906922; doi:10.1186/s13054-023-04328-6)
Supplement: Supplementary file 1 — Additional file 1. Supplemental tables and figure. Additional information for statistical methods. [file 13054_2023_4328_MOESM1_ESM.docx]

**SUPPLEMENTAL MATERIAL**

**Supplemental Table 1:** STROBE Statement—checklist of items that should be included in reports of observational studies.

**Supplemental Table 2:** Definitions of the target trial and its emulation, as reported in Dickerman et al [1].

**Supplemental Table 3:** Description of the nested trials cohort.

**Supplemental Table 4:** Multivariable Cox model for the effect of levosimendan on ECMO weaning success in the target trial emulation.

**Supplemental Figure 1:** Levosimendan administration since beginning of ECMO support.

**Supplemental File:** Additional information for statistical methods.

**Supplemental References:** list of references in supplemental materials.

**Supplemental Table 1:** STROBE Statement—checklist of items that should be included in reports of observational studies.

|  | Item No. | Recommendation | Page  No. | Relevant text from manuscript |
| --- | --- | --- | --- | --- |
| **Title and abstract** | 1 | (*a*) Indicate the study’s design with a commonly used term in the title or the abstract | 1 | Effects of levosimendan in patients undergoing extracorporeal membrane oxygenation after cardiac surgery: an emulated target trial |
|  |  | (*b*) Provide in the abstract an informative and balanced summary of what was done and what was found | 2 |  |
| Introduction | | | |  |
| Background/rationale | 2 | Explain the scientific background and rationale for the investigation being reported | 3 |  |
| Objectives | 3 | State specific objectives, including any prespecified hypotheses | 3 | We therefore sought to evaluate whether levosimendan administration could improve VA-ECMO weaning or have an impact on mortality in a large population of patients with post-cardiotomy cardiogenic shock |
| Methods | | | |  |
| Study design | 4 | Present key elements of study design early in the paper | 4, 6-7 | This is a single-center cohort study with retrospective data collection (…) We thus emulated an hypothetical target trial as proposed by Hernán et al[21-23] in which patients were randomly assigned to levosimendan administration or no levosimendan administration |
| Setting | 5 | Describe the setting, locations, and relevant dates, including periods of recruitment, exposure, follow-up, and data collection | 4-7 | We enrolled all patients admitted to the surgical Intensive Care Unit (ICU) at the Cardiology Institute of La Pitié-Salpêtrière University Hospital (Paris, France) between January 2016 and December 2019. (…)  Patients (...) were followed from baseline (...) until death or 1-year after ICU admission |
| Participants | 6 | (*a*) *Cohort study*—Give the eligibility criteria, and the sources and methods of selection of participants. Describe methods of follow-up  *Case-control study*—Give the eligibility criteria, and the sources and methods of case ascertainment and control selection. Give the rationale for the choice of cases and controls  *Cross-sectional study*—Give the eligibility criteria, and the sources and methods of selection of participants | 4-5 | The main inclusion criterion was the presence of mechanical circulatory support with VA-ECMO in the setting of cardiovascular surgery. We excluded patients who were less than 18 years old, those who had other types of mechanical circulatory support, those who had ECMO in a non-surgical context and who started ECMO support before surgery.(…) Baseline covariates and follow-up of patients were collected retrospectively by medical record consultation and computer extraction of biological data. |
|  |  | (*b*) *Cohort study*—For matched studies, give matching criteria and number of exposed and unexposed  *Case-control study*—For matched studies, give matching criteria and the number of controls per case | NA |  |
| Variables | 7 | Clearly define all outcomes, exposures, predictors, potential confounders, and effect modifiers. Give diagnostic criteria, if applicable | 5, 7-8 | The primary outcome was time to successful weaning from VA-ECMO within 30 days. Successful ECMO weaning was defined as follows: ECMO removal within 30 days in a patient being alive, and without one of the following events occurring in the 30 days after ECMO removal: death, repeat ECMO, need for another mechanical circulatory device or heart transplantation (ECMO weaning failure after ECMO removal). (…) All patients in the levosimendan group received a continuous infusion of 0.2µg/kg/min over 24 hours (12.5mg of Levosimendan diluted in 50ml of 0.9% NaCl) administered as a single dose without an initial bolus of levosimendan (...)  The effect of levosimendan on the primary outcome was then estimated on pooled nested trials data using a univariable and a multivariable Cox model. (…) Covariates entered into the model were as follows: age, sex, SAPS II at ICU admission, history of chronic heart failure, previous sternotomy, hypertension, diabetes mellitus, type of acute heart failure, indication for surgery, chronic kidney disease, BMI, SOFA score, lactatemia, ECMO blood flow rate indexed to body surface area, and VIS. SOFA score, ECMO output, lactatemia, and VIS were measured at baseline of each nested trial, all other covariates were measured at ICU admission or at beginning of ECMO support. |
| Data sources/ measurement | 8* | For each variable of interest, give sources of data and details of methods of assessment (measurement). Describe comparability of assessment methods if there is more than one group | 5-6 | Baseline covariates and follow-up of patients were collected retrospectively by medical record consultation and computer extraction of biological data. The following covariates were collected at baseline: age, height, weight (with calculation of body surface area according to Boyd's formula), sex, Simplified Acute Physiology Score (SAPS) II at ICU admission, history of chronic left heart failure, hypertension, previous sternotomy, chronic kidney disease, diabetes mellitus, indication for surgery and type of acute heart failure at beginning of ECMO support. The following covariates were collected daily during the ICU stay: creatinine, total bilirubin, platelet count, ECMO output, ongoing renal replacement therapy (RRT), lactate, total epinephrine dose, total norepinephrine dose, and total dobutamine dose. |
| Bias | 9 | Describe any efforts to address potential sources of bias | 6-8 | This methodological strategy (the emulated target trial framework) makes coincide the time of eligibility criteria assessment, treatment assignment and starting of follow-up, thus removing immortal time bias related to the delay between ECMO initiation and levosimendan administration. (…)  The effect of levosimendan on the primary outcome was then estimated on pooled nested trials data using a univariable and a multivariable Cox model, as the decision to prescribe levosimendan was at the discretion of the medical team in this cohort. |
| Study size | 10 | Explain how the study size was arrived at | NA |  |

Continued on next page

| Quantitative variables | 11 | Explain how quantitative variables were handled in the analyses. If applicable, describe which groupings were chosen and why | Supplemental File | IGS2, Age, BMI, lactate and SOFA score were binarized, as they did not respect loglinerarity as a continuous variable in the Cox model. |
| --- | --- | --- | --- | --- |
| Statistical methods | 12 | (*a*) Describe all statistical methods, including those used to control for confounding | 8 | The effect of levosimendan on the primary outcome was then estimated on pooled nested trials data using (…) a multivariable Cox model to adjust for confounding bias |
|  |  | (*b*) Describe any methods used to examine subgroups and interactions | 9 | We performed subgroup analyses of the primary outcome with tests for treatment-by-subgroup interaction. The interaction terms tested were: time to treatment assignment (…) (until day 2 or after day 2), time of ECMO implantation (in the operating room or in the ICU), type of acute heart failure (right or left ventricular heart failure or right and left ventricular heart failure), and indication for surgery (heart transplantation versus others). The interaction was tested with the Wald test. |
|  |  | (*c*) Explain how missing data were addressed | 9 and Supplemental File |  |
|  |  | (*d*) *Cohort study*—If applicable, explain how loss to follow-up was addressed  *Case-control study*—If applicable, explain how matching of cases and controls was addressed  *Cross-sectional study*—If applicable, describe analytical methods taking account of sampling strategy | 9 and Supplemental File | No patient was lost to follow-up for the primary outcome and 30-day mortality. (…) the analyses were performed on complete cases for 1-year mortality. |
|  |  | (*e*) Describe any sensitivity analyses | 9 | First, we performed a sensitivity analysis excluding patients from 2016, (…) , to assess if change in clinical practice may have affected the per protocol estimate of the primary outcome. Then, we used Fine and Gray’s competing risks model to estimate the subdistribution hazard ratio for ECMO weaning success (sHR) [32]. |
| Results | | | | |
| Participants | 13* | (a) Report numbers of individuals at each stage of study—eg numbers potentially eligible, examined for eligibility, confirmed eligible, included in the study, completing follow-up, and analysed | Figure 1 and Figure 3 |  |
|  |  | (b) Give reasons for non-participation at each stage | Figure 1 and Figure 3 |  |
|  |  | (c) Consider use of a flow diagram | Figure 1 and Figure 3 |  |
| Descriptive data | 14* | (a) Give characteristics of study participants (eg demographic, clinical, social) and information on exposures and potential confounders | Table 1 and Supplemental Table 3 |  |
|  |  | (b) Indicate number of participants with missing data for each variable of interest | Table 1 and Supplemental Table 3 |  |
|  |  | (c) *Cohort study*—Summarise follow-up time (eg, average and total amount) | 9, 11 | No patient was lost to follow-up for the primary outcome and 30-day mortality. (…) After one year, 7 patients were lost to follow-up. |
| Outcome data | 15* | *Cohort study*—Report numbers of outcome events or summary measures over time | Figure 2 |  |
|  |  | *Case-control study—*Report numbers in each exposure category, or summary measures of exposure | NA |  |
|  |  | *Cross-sectional study—*Report numbers of outcome events or summary measures | NA |  |
| Main results | 16 | (*a*) Give unadjusted estimates and, if applicable, confounder-adjusted estimates and their precision (eg, 95% confidence interval). Make clear which confounders were adjusted for and why they were included | 11, Supplemental Table 4 | Regarding the time to successful ECMO weaning, no statistically significant association with levosimendan treatment was found in in univariate analysis (HR= 1.34, 95% confidence interval CI95 [0.92;1.96], p=0.122 (...)Likewise, in multivariate analysis, levosimendan was not associated with successful ECMO weaning (HR= 0.91, CI95 [0.57;1.45], p=0.659) |
|  |  | (*b*) Report category boundaries when continuous variables were categorized | Supplemental File |  |
|  |  | (*c*) If relevant, consider translating estimates of relative risk into absolute risk for a meaningful time period | NA |  |

Continued on next page

| Other analyses | 17 | Report other analyses done—eg analyses of subgroups and interactions, and sensitivity analyses | 11-12, Table 2 | The effect of levosimendan on ECMO weaning success was also non-significant after exclusion of patients from 2016, (adjusted HR of 0.73, CI95 [0.44;1.21], per protocol estimate). Sensitivity analysis with the subdistribution hazard competing risk model found in univariable analysis a statistically significant association between levosimendan treatment and successful ECMO weaning (sHR = 1.60 CI95 [1.1;2.32], p= 0.014), that was not significant anymore after adjustment on confounding factors (sHR = 1.01, CI95 [0.64;1.59], p= 0.961). (...)  Subgroup analyses according to the timing of ECMO implantation, timing of treatment assignment (…), single or bi-ventricular failure, ECMO-implantation timing (…), indication for surgery and RTT showed no significant difference between patients receiving levosimendan or not (Table 2). |
| --- | --- | --- | --- | --- |
| Discussion | | | | |
| Key results | 18 | Summarise key results with reference to study objectives | 13 | Our study found no benefit of levosimendan administration on ECMO-weaning success and mortality in patients with refractory postcardiotomy cardiogenic shock. |
| Limitations | 19 | Discuss limitations of the study, taking into account sources of potential bias or imprecision. Discuss both direction and magnitude of any potential bias | 15-16 | the study gets some limitations due to its observational nature: data were collected retrospectively, available information restrain the possibilities to define eligibility criteria, and blinding could not be emulated (…) Second, missingness was present. (…) Third, (…) unmeasured confounders can still explain the observed estimates that would have been absent in a randomized trial. (…) Fourth, despite the relatively large size of our cohort, (…) the power of analyses was still restricted by the limited number of observations that received levosimendan. (…)  Fifth, the dosage of levosimendan administered in our study (0.2µg/kg/min) is in accordance with the label dosage (maximum dosage). To our knowledge, the only pharmacological study of levosimendan under ECMO by Sangalli et al. [43] found a benefit of levosimendan on endothelial function and cardiac output for doses twice as low as in our study. In our study, the maximum dosage was associated with hypotension requiring vasopressor support. Thus, the ideal dose may be lower.  (…)  Finally, we could not control timing of administration of levosimendan as in a randomized trial, and the time between ECMO implantation and levosimendan administration was a median of 5 days [3-6]. |
| Interpretation | 20 | Give a cautious overall interpretation of results considering objectives, limitations, multiplicity of analyses, results from similar studies, and other relevant evidence | 13-16 |  |
| Generalisability | 21 | Discuss the generalisability (external validity) of the study results | 16-17 | Sixth, the main cause of refractory post-cardiotomy cardiogenic shock in our study was acute primary graft dysfunction. Therefore, the results of our study should be cautiously applied to other tertiary centers with more prevalent subpopulations at higher risk of failure of ECMO weaning (valvular, aortic and combined surgery) than patients with heart transplantation [2].  The time between ECMO implantation and levosimendan administration was a median of 5 days [3-6]. (…) However, the delay between ECMO implantation and levosimendan infusion in the two studies finding a benefit of levosimendan on ECMO-weaning were 1 day (Distelmaier et al) and 3.2 days (Vally et al) compared to 6.6 days in the study conducted by Guilherme et al., thus very similar to our study, and where no significant association of levosimendan and ECMO weaning was found. |
| Other information | |  | | |
| Funding | 22 | Give the source of funding and the role of the funders for the present study and, if applicable, for the original study on which the present article is based | 26 |  |

**Supplemental Table 2:** Definitions of the target trial and its emulation, as reported in Dickerman et al [1].

| **Protocol component** | **Target trial specification** | **Target trial emulation** |
| --- | --- | --- |
| Eligibility criteria | Inclusion criteria   - Age ≥ 18 - Mechanical circulatory support with VA-ECMO - Admission after cardiovascular surgery - Admitted to the surgical Intensive Care Unit at the Cardiology Institute of La Pitié-Salpêtrière University Hospital   Non-inclusion criteria   - Veno-venous ECMO - Admission in non-surgical context - ECMO support before surgery - No previous levosimendan administration for ECMO weaning | Same as for the target trial |
| Treatment strategies | (1) Administration of levosimendan for ECMO weaning: Continuous infusion of 0.2µg/kg/min over 24 hours (12.5mg of levosimendan diluted in 50ml of 0.9% NaCl) administered as a single dose without an initial bolus of levosimendan  (2) No administration of levosimendan for ECMO weaning | Same as for the target trial  We defined the date of levosimendan administration as the date of initiation of the infusion |
| Treatment assignment | Participants will be randomly assigned to a treatment strategy at baseline. | Participants were assigned to the treatment strategy according to their baseline data on the ECMO day  The analysis was adjusted for baseline confounders to simulate randomization |
| Outcomes | Time to ECMO weaning success  Mortality at 30-day and 1-year | Same as for the target trial |
| Follow-up | Participants will be followed from baseline until death, 1-year after ICU admission or loss to follow-up whichever occurs first | Same as for the target trial |
| Causal contrasts | Intention-to-treat and per protocol effect | Analog of per-protocol effect as main causal contrast, analog of intention-to-treat effect as a secondary analysis |
| Statistical analysis | Intention-to-treat and per-protocol analyses  Subgroups analyses on ECMO insertion timing (Operating room or ICU), monoventricular or biventricular heart failure, and indication for surgery (heart transplantation versus others) | Analog of intention-to-treat and per protocol analyses.  Same subgroups analyses and additional subgroup analysis on treatment assignment up to ECMO day 2 or after day 2 |

*Abbreviations:* VA-ECMO, veno-arterial extracorporeal membrane oxygenation; ICU, intensive care unit.

**Supplemental Table 3:** Description of the nested trials cohort.

|  | | | **Levosimendan** | | **p-value** |
| --- | --- | --- | --- | --- | --- |
|  |  |  | **No (n=1369)** | **Yes (n=65)** |  |
| **On admission** | | | | | |
| Age (years) | Median [Q1-Q3] | | 62 [54-69] | 62 [58-72] | 0.355 |
| Sex | Female | | 530 (39%) | 22 (34%) | 0.431 |
| BMI (Kg/m^2^) | Median [Q1-Q3] | | 27 [23-31] | 25 [22-28] | **0.011** |
|  | N (NA) | | 1362 (7) | 65 (0) |  |
| Medical conditions | Hypertension | | 781 (57%) | 36 (55%) | 0.791 |
|  | Diabetes | | 305 (22%) | 20 (31%) | 0.1102 |
|  | Dyslipidemia | | 481 (35%) | 26 (40%) | 0.423 |
|  | COPD | | 161 (12%) | 6 (9%) | 0.535 |
|  | Peripheral vascular disease |  | 146 (11%) | 9 (14%) | 0.406 |
|  |  | N (NA) | 1358 (11) | 64 (1) |  |
|  | Chronic heart failure |  | 1029 (78%) | 49 (77%) | 0.7143 |
|  |  | N (NA) | 1315 (51) | 64 (1) |  |
|  | Previous sternotomy | | 522 (38%) | 20 (31%) | 0.2318 |
|  | Chronic kidney disease | | 367 (27%) | 18 (28%) | 0.8903 |
| SAPS II | Median [Q1-Q3] | | 34.1 [4.9-57] | 55.5 [12.6-64.5] | **0.001** |
|  | N (NA) | | 1359 (10) | 64 (1) |  |
| Euroscore | Median [Q1-Q3] | | 4.1 [2-11.6] | 6.4 [2-25] | 0.074 |
|  | N (NA) | | 1412 (56) | 64 (4) |  |
| Type of surgery | CABG | | 99 (7%) | 10 (15%) | 0.073 |
|  | Valvular surgery | | 434 (32%) | 18 (28%) |  |
|  | Combined valvular and CABG | | 149 (11%) | 4 (6%) |  |
|  | Heart transplant | | 501 (37%) | 26 (40%) |  |
|  | LVAD | | 3 (0.2%) | 1 (2%) |  |
|  | TAVI | | 2 (0.1%) | 0 (0%) |  |
|  | Other | | 181 (13%) | 6 (9%) |  |
| **In the operating room** | | | | | |
| CPB duration (minutes) | Median [Q1-Q3] | | 143 [110-197] | 136 [98-190] | 0.236 |
|  | N (NA) | | 1361 (8) | 65 (0) |  |
| Aortic cross clamping (minutes) | Median [Q1-Q3] | | 85 [66-129] | 77 [55-127] | 0.274 |
|  | N (NA) | | 1291 (78) | 62 (3) |  |
| RBC (units) | Median [Q1-Q3] | | 2 [0-6] | 2 [0-5] | 0.257 |
|  | N (NA) | | 1337 (32) | 64 (1) |  |
| Frozen plasma (units) | Median [Q1-Q3] | | 3 [0-7] | 2 [0-6] | 0.101 |
|  | N (NA) | | 1337 (32) | 64 (1) |  |
| Platelets (units) | Median [Q1-Q3] | | 1 [0-1] | 1 [0-1] | 0.449 |
|  | N (NA) | | 1337 (32) | 64 (1) |  |
| Fibrinogen | Yes | | 466 (35%) | 20 (31%) | 0.483 |
|  | N (NA) | | 1349 (20) | 64 (1) |  |
| **At ECMO implantation (unless specified)** | | | | | |
| Timing | Intraoperative | | 1052 (77%) | 51 (78%) | 0.762 |
|  | Postoperative | | 317 (23%) | 14 (22%) |  |
| Ventricular failure at implantation | Left | | 237 (17%) | 22 (34%) | **0.002** |
|  | Right | | 148 (11%) | 8 (12%) |  |
|  | Right and left | | 984 (72%) | 35 (54%) |  |
| ECMO site | Central | | 136 (10%) | 3 (5%) | 0.157 |
| SOFA* | Median [Q1-Q3] | | 12 [11-13] | 11 [10-12] | **0.005** |
|  | N (NA) | | 1063 (306) | 47 (18) |  |
| Daily total dose of epinephrine (mg)* | Mean (SD) | | 4.7 (15.1) | 1.3 (6.2) | **0.032** |
|  | N (NA) | | 1333 (36) | 65 (0) |  |
| Daily total dose of norepinephrine (mg)* | Median [Q1-Q3] | | 0.6 [0-19.8] | 0.6 [0-10.2] | 0.232 |
|  | N (NA) | | 1334 (35) | 65 (0) |  |
| Daily total dose of dobutamine (mg)* | Median [Q1-Q3] | | 186.6 [0-593.3] | 0 [0-360.1] | **0.021** |
|  | N (NA) | | 1334 (35) | 65 (0) |  |
| VIS* | Median [Q1-Q3] | | 8.8 [4-25.9] | 5.9 [0.8-12.2] | **0.002** |
|  | N (NA) | | 1331 (38) | 65 (0) |  |
| Lactate* | Median [Q1-Q3] | | 1.7 [1.1-2.7] | 1.4 [1.1-2] | **0.013** |
|  | N (NA) | | 1293 (76) | 63 (2) |  |
| ECMO output* (L/min/m2) | Median [Q1-Q3] | | 1.8 [1.4-2.1] | 1.7 [1.3-2] | 0.186 |
|  | N (NA) | | 1312 (57) | 64 (1) |  |
| Impella® | Yes | | 96 (7%) | 4 (6%) | 1.000 |
| Delay between CPB cessation and ECMO implantation (hours) | Median [Q1-Q3] | | 0 [0-0] | 0 [0-0] | 0.6931 |
|  | N (NA) | | 1310 (59) | 64 (1) |  |
| Delay between ECMO implantation and levosimendan infusion (days) | Median [Q1-Q3] | | - | 5 [3-6] |  |
| **Events under ECMO (unless specified)** | IABP | | 583 (43%) | 27 (42%) | 0.868 |
|  | Digestive bleeding | | 314 (23%) | 13 (20%) | 0.581 |
|  | Acute mesenteric ischemia |  | 258 (19%) | 5 (8%) | **0.023** |
|  |  | N (NA) | 1350 (19) | 64 (1) |  |
|  | Acute kidney injury |  | 1117 (82%) | 44 (68%) | **0.004** |
|  |  | N (NA) | 1361 (8) | 65 (0) |  |
|  | Extra-renal replacement therapy* | | 473 (34%) | 18 (28%) | 0.255 |
|  | Stroke | | 185 (14%) | 13 (20%) | 0.139 |
|  | Mediastinitis | | 183 (13%) | 10 (15%) | 0.642 |
|  | Bacteremia | | 568 (41%) | 27 (42%) | 0.994 |
|  | Septic shock | | 868 (63%) | 36 (55%) | 0.191 |
|  | Hemorrhagic shock | | 592 (43%) | 35 (54%) | 0.092 |
|  | Cardiac arrest | | 196 (14%) | 8 (12%) | 0.651 |
|  | Acute coronary syndrome | | 91 (7%) | 1 (2%) | 0.120 |
|  | VAP | | 1067 (78%) | 47 (72%) | 0.287 |
|  | Limb ischemia |  | 195 (15%) | 8 (13%) | 0.678 |
|  |  | N (NA) | 1337 (32) | 63 (2) |  |
|  | Scarpa infection |  | 204 (15%) | 8 (13%) | 0.578 |
|  |  | N (NA) | 1336 (33) | 63 (2) |  |
|  | Surgical revision of the ECMO implantation site |  | 255 (19%) | 10 (16%) | 0.502 |
|  |  | N (NA) | 1343 (26) | 64 (1) |  |

*Abbreviations:* BMI, body mass index; CABG, coronary artery bypass graft; CPB, cardiopulmonary bypass; COPD, chronic obstructive pulmonary disease; ECMO, extracorporeal membrane oxygenation; IABP, intra-aortic balloon pump; LVAD, left ventricular assist device; NA, not available (missing data); Q1, first quartile; Q3, third quartile; RBC, red blood cells; SAPS II, Simplified Acute Physiology Score II; SOFA, Sequential Organ Failure Assessment; TAVI, transcatheter aortic valve implantation; VAP, ventilator associated pneumonia; VIS, vasoactive inotropic score. For covariates which the median was null in both groups, they were described with their mean and standard deviation.*The values reported for these covariates are the ones at baseline of the nested trial, and not the ones at ECMO implantation or under ECMO.

**Supplemental Table 4:** Multivariable Cox model for the effect of levosimendan on ECMO weaning success in the target trial emulation.

| **Covariate** | **Hazard ratio for ECMO weaning success [CI95]** | **p-value** |
| --- | --- | --- |
| Levosimendan | 0.91, [0.57;1.45] | 0.686 |
| SAPS II | 0.83, [0.37;1.87] | 0.661 |
| Age | 1.17, [0.66;2.06] | 0.589 |
| BMI | 0.35, [0.15;0.81] | 0.014 |
| Chronic heart failure | 0.57, [0.27;1.20] | 0.138 |
| SOFA score | 0.76, [0.52;1.11] | 0.156 |
| Chronic kidney disease | 0.92, [0.47;1.78] | 0.800 |
| Hypertension | 0.51, [0.28;0.94] | 0.032 |
| Diabetes mellitus | 1.50, [0.80;2.83] | 0.207 |
| Indication for surgery, days 0-9 | 3.41, [1.50;7.77] | 0.002 |
| Indication for surgery, after day 9 | 0.39, [0.04;4.19] |  |
| Sex, days 0-9 | 1.04, [0.60;1.81] | <0.0001 |
| Sex, after day 9 | 1.32, [0.18;9.61] |  |
| VIS, days 0-3 | 0.80, [0.68;0.95] | 0.034 |
| VIS, after day 3 | 0.96, [0.89;1.04] |  |
| Lactate, days 0-2 | 0.39, [0.25;0.61] | 0.0002 |
| Lactate, after day 2 | 0.79, [0.54;1.15] |  |
| ECMO output, days 0-1 | 0.16, [0.09;0.28] | <0.0001 |
| ECMO output, days 2-4 | 0.59, [0.34;1.03] |  |
| ECMO output, after day 4 | 0.81, [0.44;1.51] |  |
| Previous sternotomy, days 0-7 | 0.83, [0.46;1.52] | 0.204 |
| Previous sternotomy, after day 7 | 0.17, [0.03;1.19] |  |
| Acute heart failure (day 0) | 0.63, [0.33;1.20] | 0.027 |
| Acute heart failure (day 2) | 0.86, [0.46;1.60] |  |
| Acute heart failure (day 7) | 1.88, [0.76;4.62] |  |
| Acute heart failure (day 10) | 3.00, [0.92;9.82] |  |

All covariates were measured at ICU admission, beginning of ECMO support or baseline of each nested trial. However, some covariates were transformed to get a time-varying effect (and an associated time-varying value of hazard ratio) due to violation of proportional hazards assumption. The shown p-values are from the Wald test, with the null hypothesis that all of the coefficients in the model of a given covariate are equal to zero.

*Abbreviations:* BMI, body mass index; CI95, 95% confidence interval; ECMO, extracorporeal membrane oxygenation; ICU, intensive care unit; SAPS II, Simplified Acute Physiology Score II; SOFA, Sequential Organ Failure Assessment; VIS, vasoactive inotropic score.

**Supplemental Figure 1:** Levosimendan administration since beginning of ECMO support.

**Supplemental File:** Additional information for statistical methods.

1. **Target trial emulation and construction of the nested trials cohort**

Target trial emulation is a methodological framework to conduct observational studies and avoid biases in their design and their analyses. First, the target trial must be defined, that is the trial that would have been conducted in the interventional settings. All the components of the protocol of this target trial must be specified, as in the supplemental Table 2. Then all the elements of the protocol are emulated with observational data. This point-by-point comparison of key components of the protocol makes easier the identification of potential biases and help to adapt the statistical strategy to avoid the preventable biases[2].

In this study, we chose to emulate the target trial sequentially, as a series of nested trials. Each day of VA-ECMO support, if a patient was eligible to our target trial (the patient was still with VA-ECMO support, alive and did not receive levosimendan in the previous days since beginning of VA-ECMO support), a copy of the patient was created. For each copy of patient, we recorded the daily status for exposure to levosimendan, and other covariates (lactate, ECMO output, ongoing RRT, VIS and SOFA score). Finally, this copy of patient was kept in the analysis if the comparison of the target trial was feasible, or if on that particular day since beginning of VA-ECMO support at least one patient received levosimendan and could be compared to patients that did not. To avoid immortal time bias, we needed to make coincide the assessment of eligibility criteria, the treatment assignment and the beginning of follow-up for the copy of the patient (or the time zero). The follow-up of a given copy of patient was then reinitialized at the day of the nested trial. For example, at day 3 since beginning of ECMO support, the follow-up of a patient still eligible to the nested trial was the follow-up since beginning of VA-ECMO support minus three days. After emulating all the possible trials, the copies of patients were pooled and the analysis was performed on the stacked database. Therefore, each copy of patient in the pooled database had daily information for exposure, and a daily value for time-varying covariates, that were introduced in the models. As a patient could participate to more than one nested trial, we estimated robust variances by bootstrap (1000 iterations). This method of the nested trials have been published elsewhere and already applied to various clinical questions by several teams [1,3–6]. For the construction of duplicated observations, more information is also available in Figure 1.

1. **List of covariates included in the multivariable models for ECMO weaning success**

|  | **Transformation** | **Assessment** |
| --- | --- | --- |
| Levosimendan |  | Baseline of nested trial |
| SAPS II | <40 or ≥40 | ICU admission |
| Age | < 60 or ≥ 60 years | ICU admission |
| BMI | ≤ 30 or >30 kg/m^2^ | ICU admission |
| Chronic heart failure |  | ICU admission |
| SOFA score | <12 or ≥ 12 | Baseline of nested trial |
| Chronic kidney disease |  | ICU admission |
| Hypertension |  | ICU admission |
| Diabetes mellitus |  | ICU admission |
| Indication for surgery, days 0-9 | Heart transplantation versus others | ICU admission |
| Indication for surgery, after day 9 | Heart transplantation versus others | ICU admission |
| Sex, days 0-9 | Female versus Male | ICU admission |
| Sex, after day 9 | Female versus Male | ICU admission |
| VIS, days 0-3 | Continuous, per 10 units | Baseline nested trial |
| VIS, after day 3 | Continuous, per 10 units | Baseline nested trial |
| Lactate, days 0-2 | < 2 or ≥ 2 | Baseline of nested trial |
| Lactate, after day 2 | < 2 or ≥ 2 | Baseline of nested trial |
| ECMO output, days 0-1 | Continuous, indexed to body surface area | Baseline of nested trial |
| ECMO output, days 2-4 | Continuous, indexed to body surface area | Baseline of nested trial |
| ECMO output, after day 4 | Continuous, indexed to body surface area | Baseline of nested trial |
| Previous sternotomy, days 0-7 |  | ICU admission |
| Previous sternotomy, after day 7 |  | ICU admission |
| Acute heart failure | Monoventricular versus biventricular dysfunction | Beginning of ECMO support |
| Acute heart failure, time-varying effect | Acute heart failure x time since the beginning of the emulated trial | Beginning of ECMO support |

Note: IGS2, Age, BMI, lactate and SOFA score were binarized, as they did not respect loglinerarity as a continuous variable in the models.

1. **List of covariates included in the multivariable logistic regression models for 30-day and 1-year mortality.**

|  | **Transformation** | **Assessment** |
| --- | --- | --- |
| Levosimendan |  | Baseline of nested trial |
| Age | < 60 or ≥ 60 years | ICU admission |
| Sex | Female versus Male | ICU admission |
| SAPS II | <40 or ≥40 | ICU admission |
| Chronic heart failure |  | ICU admission |
| Acute heart failure | Monoventricular versus biventricular dysfunction | Beginning of ECMO support |
| Lactate | < 2 or ≥ 2 | Baseline of nested trial |
| SOFA score | <12 or ≥ 12 | Baseline of nested trial |
| ECMO output | Indexed to body surface area, < 2 or ≥ 2 | Baseline of nested trial |
| VIS | < 10 or ≥ 10 | Baseline of nested trial |
| Indication for surgery | Heart transplantation versus others | ICU admission |
| Chronic kidney disease |  | ICU admission |
| Hypertension |  | ICU admission |
| BMI | ≤ 30 or >30 kg/m^2^ | ICU admission |
| Previous sternotomy |  | ICU admission |
| Diabetes mellitus |  | ICU admission |

Note: Age, SAPS II, BMI, lactate, SOFA score, VIS, and ECMO flow rate were binarized, as they did not respect loglinerarity as a continuous variable in the logistic regression model.

1. **Missing Data handling**

Missing data for all covariates (except for age and gender for which they were no missing-data) were handled with an approach that considers the time-dependency of the covariates in the imputation model [7]. Briefly, this method is based on multiple imputations by chained equations (MICE package in R v3.14.0, [8]) and introduces in the imputation matrix the values of the time-dependent covariates at a given day and the values of these same covariates for the four previous days, as well as the incidence of the primary outcome. Ten imputations were performed, and the Rubin's rule was applied to compute model coefficients [9]. Due to lack of observations at the last timepoints (day 29 to day 40), we were not able to impute the time-dependent covariates with this method, the values were replaced by the mean value of each covariate. Of note, imputed data after day 28 were used only in the Cox model used to estimate weights for inverse probability of censoring weighting, as we did not introduce covariates with time-varying values (but only with time-varying effects) in the Cox or Fine and Gray models for estimation of the hazard ratio of ECMO weaning success, and the last nested trial was at day 20. No patient was lost to follow-up for the primary outcome, and the additional 30-day follow-up to qualify the event was available for all participants. Due to a low proportion of missingness (after one year, 7 patients were lost to follow-up) and its exploratory nature, the analyses were performed on complete cases for 1-year mortality.

1. **Inverse probability of censoring weighting**

Our primary analysis was performed on the per protocol dataset, in which patients of the control group who started levosimendan at a later timepoint in a given nested trial were censored at the date of receiving levosimendan. Thus, the design introduced informative censoring. To limit the induced selection bias, a strategy of inverse probability of censoring weighting was implemented. Weights were estimated by a multivariable Cox model that predicts the probability of informative censoring for each observation in the control group. We introduced in this model time-varying covariates: daily lactate, biological SOFA, ECMO output indexed to body surface and VIS; and covariates from ICU baseline assessment: age (categorized as less than or greater than 60 years); gender; SAPS II; BMI (categorized as less than or greater than 30 kg/m^2^); history of diabetes mellitus, chronic heart failure, chronic kidney disease, hypertension or heart surgery; indication for surgery during ICU stay (categorized as heart transplantation versus other surgeries) and the day of the nested trial [10,11]. Stabilized weights were computed for the observations of the control group and introduced in the Cox model used to estimate the cause specific hazard ratio for ECMO weaning success.

**Supplemental References:** list of references in supplemental materials.

[1] Dickerman BA, García-Albéniz X, Logan RW, Denaxas S, Hernán MA. Avoidable flaws in observational analyses: an application to statins and cancer. Nat Med 2019;25:1601–6. https://doi.org/10.1038/s41591-019-0597-x.

[2] Hernán MA, Sauer BC, Hernández-Díaz S, Platt R, Shrier I. Specifying a target trial prevents immortal time bias and other self-inflicted injuries in observational analyses. J Clin Epidemiol 2016;79:70–5. https://doi.org/10.1016/j.jclinepi.2016.04.014.

[3] Rossides M, Kullberg S, Di Giuseppe D, Eklund A, Grunewald J, Askling J, et al. Infection risk in sarcoidosis patients treated with methotrexate compared to azathioprine: A retrospective ‘target trial’ emulated with Swedish real‐world data. Respirology 2021;26:452–60. https://doi.org/10.1111/resp.14001.

[4] Peterson RG, Xiao R, Katcoff H, Fisher BT, Weiss PF. Effect of first-line biologic initiation on glucocorticoid exposure in children hospitalized with new-onset systemic juvenile idiopathic arthritis: emulation of a pragmatic trial using observational data. Pediatr Rheumatol 2021;19:109. https://doi.org/10.1186/s12969-021-00597-z.

[5] the STOP-COVID Investigators, Shaefi S, Brenner SK, Gupta S, O’Gara BP, Krajewski ML, et al. Extracorporeal membrane oxygenation in patients with severe respiratory failure from COVID-19. Intensive Care Med 2021;47:208–21. https://doi.org/10.1007/s00134-020-06331-9.

[6] Hajage D, Combes A, Guervilly C, Lebreton G, Mercat A, Pavot A, et al. Extracorporeal Membrane Oxygenation for Severe Acute Respiratory Distress Syndrome associated with COVID-19: An Emulated Target Trial Analysis. Am J Respir Crit Care Med 2022:rccm.202111-2495OC. https://doi.org/10.1164/rccm.202111-2495OC.

[7] Murad H, Dankner R, Berlin A, Olmer L, Freedman LS. Imputing missing time-dependent covariate values for the discrete time Cox model. Stat Methods Med Res 2020;29:2074–86. https://doi.org/10.1177/0962280219881168.

[8] Buuren S van, Groothuis-Oudshoorn K. **mice** : Multivariate Imputation by Chained Equations in *R*. J Stat Softw 2011;45. https://doi.org/10.18637/jss.v045.i03.

[9] Rubin DB, editor. Multiple Imputation for Nonresponse in Surveys. Hoboken, NJ, USA: John Wiley & Sons, Inc.; 1987. https://doi.org/10.1002/9780470316696.

[10] Gran JM, Røysland K, Wolbers M, Didelez V, Sterne JAC, Ledergerber B, et al. A sequential Cox approach for estimating the causal effect of treatment in the presence of time-dependent confounding applied to data from the Swiss HIV Cohort Study. Stat Med 2010;29:2757–68. https://doi.org/10.1002/sim.4048.

[11] Maringe C, Benitez Majano S, Exarchakou A, Smith M, Rachet B, Belot A, et al. Reflection on modern methods: trial emulation in the presence of immortal-time bias. Assessing the benefit of major surgery for elderly lung cancer patients using observational data. Int J Epidemiol 2020;49:1719–29. https://doi.org/10.1093/ije/dyaa057.
